# Operating room efficiency in a low resource setting: a pilot study from a large tertiary referral center in Ethiopia

**DOI:** 10.1186/s13037-021-00314-5

**Published:** 2022-01-07

**Authors:** Samuel Negash, Endale Anberber, Blen Ayele, Zeweter Ashebir, Ananya Abate, Senait Bitew, Miliard Derbew, Thomas G. Weiser, Nichole Starr, Tihitena Negussie Mammo

**Affiliations:** 1 Department of Surgery, Addis Ababa University, Addis Ababa, Ethiopia; 2 Department of Anesthesia, Addis Ababa University, Addis Ababa, Ethiopia; 3 Lifebox Foundation, London, UK; 4 Department of Surgery, University of Edinburgh, Edinburgh, UK; 5 Department of Surgery, Stanford University, Stanford, USA; 6 Department of Surgery, University of California San Francisco, San Francisco, USA

**Keywords:** Operating room, Efficiency, Utilization, Cancellation rate, Turnover time, Start time delay

## Abstract

**Background:**

The operating room (OR) is one of the most expensive areas of a hospital, requiring large capital and recurring investments, and necessitating efficient throughput to reduce costs per patient encounter. On top of increasing costs, inefficient utilization of operating rooms results in prolonged waiting lists, high rate of cancellation, frustration of OR personnel as well as increased anxiety that negatively impacts the health of patients. This problem is magnified in developing countries, where there is a high unmet surgical need. However, no system currently exists to assess operating room utilization in Ethiopia.

**Methodology:**

A prospective study was conducted over a period of 3 months (May 1 to July 31, 2019) in a tertiary hospital. Surgical case start time, end time, room turnover time, cancellations and reason for cancellation were observed to evaluate the efficiency of eight operating rooms.

**Results:**

A total of 933 elective procedures were observed during the study period. Of these, 246 were cancelled, yielding a cancellation rate of 35.8%. The most common reasons for cancellation were related to lack of OR time and patient preparation (8.7% and 7.7% respectively). Shortage of facilities (instrument, blood, ICU bed) were causes of cancelation in 7.7%. Start time was delayed in 93.4% (mean 8:56 am ± 52 min) of cases. Last case completion time was early in 47.9% and delayed in 20.6% (mean 2:54 pm ± 156 min). Turnover time was prolonged in 34.5% (mean 25 min ± 49 min). Total operating room utilization ranged from 10.5% to 174%. Operating rooms were underutilized in 42.7% while overutilization was found in 14.6%.

**Conclusion:**

We found a high cancellation rate, most attributable to late start times leading to delays for the remainder of cases, and lack of preoperative patient preparation. In a setting with a high unmet burden of surgical disease, OR efficiency must be maximized with improved patient evaluation workflows, adequate OR staffing and commitment to punctual start times. We recommend future quality improvement projects focusing on these areas to increase OR efficiency.

## Background

Operating rooms (ORs) are some of the most important areas of hospitals that have significant impact on the overall picture of a hospital’s performance [1, 2]. Running an operating room is capital and labor intensive [1–4]. When OR utilization is inefficient, it leads to wastage of time and human resources, higher costs and fewer patients treated than planned. This constellation of issues results in financial losses as well as decreased patient satisfaction [5].

Furthermore, recent publications regarding global surgery have highlighted the high unmet need for safe surgery in developing countries. Lack of personnel and facility are contributors to this problem [6, 7]. However, it is also our observation that many existing operating rooms in our country are underutilized. In this case, increasing efficiency can reduce patient wait times without incurring additional cost [8].

Thus far, there is scant literature assessing OR efficiency in different areas of Ethiopia [8–11]. Additionally, all of the studies assessed focused solely on the cancellation rate. Looking into this parameter alone may lead to wrong conclusion and lack of improved outcome after intervention. Other factors affecting performance such as late starts, early finishes, long time between cases and mismatch of scheduled sessions with existing capacity were not considered but have a potentially large impact on the OR efficiency.

The aim of this study was to evaluate all five indicators of OR utilization: start time, finish time turnover time and total daily utilization, together with cancellation rate. This evaluation aimed to provide a comprehensive assessment of the overall OR efficiency and inform the subsequent planning of interventions. In addition, we hope to encourage the culture of continuous monitoring of efficiency in operatinng rooms across Ethiopia.

## Methodology

This study was undertaken at Tikur Anbessa Specialized Hospital (TASH), in Addis Ababa, Ethiopia. TASH is the main teaching hospital of the country and provides many specialized clinical services that are not available in other public or private institutions. TASH is also the largest hospital in the country with a capacity of more than 700 beds. It has 12 operating rooms which is more than any other hospital in the country.

The design was a descriptive observational study. Data was collected from eight operating rooms over a period of three months (May 1 – July 31, 2019). Surgical specialties utilizing these operating rooms included otolaryngology, urology, cardiothoracic surgery, neurosurgery, pediatric surgery, general surgery and obstetrics/gynecology. Emergency operating rooms were excluded because they function without a schedule.

Based on existing literature [12, 13] and considering standard working hours to be 8 h per day starting at 8:00 am; we set standards the five indicators in our hospital (Table 1).

**Table 1:** The five indicators of OR efficiency

**Start time**	Time the first patient of the day entered the operating room **Late start** = later than 15 min of scheduled time (after 8:15 am)
**Turnover time**	Time between one patient leaving the operating table and another patient entering (time for cleaning and preparing for next patient) **Long turnover** = time in between cases > 25 min
**Finish time**	Time the last patient of the day left the operating room **Early finish time** = 1 h before working hours (before 3:00 pm) **Late finish time** = 1 h after working hours (after 5:00 pm)
**OR utilization**	Proportion of time within the working hours in which a patient was in the operating room (does not include turnover time) **Low utilization** = less than < 75% of working hour utilized (< 6 h) **Over utilization** = more than allocated working hours (> 100% / > 8 h)
**Cancellation rate**	Day of surgery cancellation **High cancellation rate** = greater than 5%

A data collection tool was designed by the investigators and pretested with a subset of cases for ease of administration and clarity of terminology. Data were collected by trained operating room nurses. Data cleaning and analysis was done using SPSS version 23. Chi-squared tests were used to report outcomes and two-sided significance level was set to be 0.05 (5%). Ethical clearance was obtained from the research committee at the Department of Surgery, Addis Ababa University College of Health Sciences.

## Results

A total of 687 operations were performed during the study period. Most of the time, two procedures were performed in a room per day (39.6%), followed by a single procedure per day (36.5%). Additional procedures were sometimes performed with three procedures per day in 16.4% and 4 procedures per day in 7.4% of operating rooms.

Total number of scheduled elective cases were 933 of which 246 were cancelled, yielding a cancellation rate of 35.8%. The most common cause of cancellation was lack of OR time (workday ending before scheduled cases are finished). This was followed by issues related to perioperative patient preparation. Others were institutional factors, of which the lack of an ICU bed was the most common (Table 2).

**Table 2:** Reasons for cancellation

Reasons	*N* (%)
Lack of OR time	82 (8.7%)
Patient medically unfit for surgery	72 (7.7%)
Lack of ICU bed	41 (4.4%)
Lack of instruments	16 (1.7%)
Lack of blood	15 (1.6%)
Other	23 (2.5%)
Total	246 (35.8%)

The start time ranged from 7:45 am to 9:30 am. Mean start time was 8:56 am ± 52 min. The start time was delayed past 8:15am in 93.4% of the cases. In addition, the time of start for the first surgery (incision time) ranged from 8:00 am to 10:18 am with mean 9:41 am ± 60 min.

Turnover time ranged from 3 min to 2 h. Mean was 25 min ± 49 min. Turnover time was prolonged in 34.5%. The finish time ranged from 8:45 am to 11:12 pm. Mean finish time was 2:54 pm ± 156 min. The end time was early in 47.9% and delayed in 27.8%.

Mean number of surgeries per day was 1.9 ± 0.9. Mean time spent on surgery per day was 4 h ± 140 min. Remaining time was occupied by anesthesia preparation and intubation (1 h and 23 min ± 132 min), extubating and transfer of patients (42 min ± 34 min), turnovers (40 min ± 38 min). Total operating room utilization ranged from 10.5% to 174%. It was underutilized (< 6 h) in 42.7% of the cases while overutilization (> 8 h) was found in 14.6% (Table 3).

**Table 3:** Summary of the parameters of OR efficiency and comparison to predefined standards

Parameters	Result	Comparison to Predefined criteria
Start time	8:56 am ± 52 min	93.4% delayed
Turnover time	25 min ± 49 min	34.5% delayed
Finish time	2:54 pm ± 156 min	47.9% early 27.8% delayed
OR utilization	10.5%—174%	42.7% under utilized 14.6% over utilized
Cancelation rate	35.8%	30.8% higher

## Discussion

An efficient operating room should start early, finish on time, allocate minimal time for preparation between procedures and have a low rate of cancellation. Poor utilization of the operating room leads to wastage and drains resources which is very valuable in low income countries with a high unmet need for surgery [14, 15]. Additionally, it results in patient dissatisfaction and staff demoralization [14, 16].

In our study, the cancellation rate was 35.8% which is similar to the previous study on elective surgery cancellation in the same hospital (33.9%) [10]. Studies done in other major teaching hospitals in Ethiopia also had similar findings with the cancelation rate at Gondar University Hospital 33.1% [11] Hawassa Gondar University Hospital 31.6% [9], and Jimma University Hospital 23% [8]. The reasons for cancellation were also similarly related to shortage of time (over scheduling) and inadequate patient preparation. This is in contradiction to the belief that higher rate of cancellation in low income countries is unavoidable (due to resource constraints) [17].

We found turnover time was 25 min ± 49 min with delay in 34.5%. This is an acceptable figure considering the best performing operating rooms report a turnover time less than 25 min [13]. Furthermore, we have found the number of procedures done per OR each day is low (76% perform either 1 or 2 procedures per OR per day). Therefore, attempting to decrease turnover time would likely not have significant impact in this setting.

The start time was delayed in 93.4% of the cases. This may be the most important parameter as it decides the day’s surgical activity [18]. Downstream effect of the late starts can also be the cause for frequent cancellations observed in our study due to lack of OR time. Many institutions have used different strategies to improve starting time [18–20]. To implement these in our setting we need further data evaluating the reasons for delay of the first case. In addition, we found frequent delays between patient entering operating room and start of surgery (43 min ± 40 min). This needs further evaluation of anesthesia induction and surgical preparation practice.

Assessment of the total OR utilization found a wide range with a majority (57.3%) either underutilized or overutilized. The finish time was very early or late in 75.7%. Early finish times can be improved by decreasing the cancellation rate, especially those related to patient preparation. Overutilization and late finish times also need to be corrected as it leads to staff burnout and overtime costs [21]. Having a late start time can again be an important factor cascading to delay in the finish time.

## Conclusion

Overall, this study found our operating rooms are inefficient, with high cancelation rates that can be attributed to delayed start times, short working days and inadequate patient preparation. This is in contrary to the belief that inefficiency in operating rooms of low-income countries is related to lack of infrastructure. While the physical space may not be a limiting factor, human resources to adequately staff ORs and meet overtime needs, and culture change around earlier start times may ameliorate some of these challenges. In a setting such as Ethiopia where there is a large unmet burden of surgical disease, future interventions to improve the function of our operating rooms should be focused on these two areas.

## Data Availability

The datasets during and/or analysed during the current study available from the corresponding author on reasonable request.

## Abbreviations

OR: Operating room
TASH: Tikur Anbessa Specialized Hospital
